# Expression Profiles of Ligands for Activating Natural Killer Cell Receptors on HIV Infected and Uninfected CD4^+^ T Cells

**DOI:** 10.3390/v9100295

**Published:** 2017-10-12

**Authors:** Alexandra Tremblay-McLean, Julie Bruneau, Bertrand Lebouché, Irene Lisovsky, Rujun Song, Nicole F. Bernard

**Affiliations:** 1 Research Institute of the McGill University Health Center, Montréal, QC H4A 3J1, Canada; alexandra.tremblay-mclean@mail.mcgill.ca (A.T.-M.); bertrand.lebouche@mcgill.ca (B.L.); lisovsky18@gmail.com (I.L.); rujun.song@mail.mcgill.ca (R.S.); 2 Division of Experimental Medicine, McGill University, Montréal, QC H4A 3J1, Canada; 3 Départment de Médecine Familiale et Médecine D’urgence, Université de Montréal, Montréal, QC H2X 0A9, Canada; julie.bruneau.umontreal@gmail.com; 4 Centre de Recherche de Centre Hospitalier de l’Université de Montréal, Montréal, QC H2X 0A9, Canada; 5 Department of Family Medicine, McGill University, Montréal, QC H4A 3J1, Canada; 6 Chronic Viral Illness Service, McGill University Health Centre, Montréal, QC H4A 3J1, Canada; 7 Division of Clinical Immunology, McGill University Health Centre, Montréal, QC H3G 1A4, Canada

**Keywords:** HIV infection, natural killer cells, HIV-infected CD4+ T cells, activating NK cell receptor ligands

## Abstract

Natural Killer (NK) cell responses to HIV-infected CD4 T cells (iCD4) depend on the integration of signals received through inhibitory (iNKR) and activating NK receptors (aNKR). iCD4 activate NK cells to inhibit HIV replication. HIV infection-dependent changes in the human leukocyte antigen (HLA) ligands for iNKR on iCD4 are well documented. By contrast, less is known regarding the HIV infection related changes in ligands for aNKR on iCD4. We examined the aNKR ligand profiles HIV p24^+^ HIV iCD4s that maintained cell surface CD4 (iCD4^+^), did not maintain CD4 (iCD4^−^) and uninfected CD4 (unCD4) T cells for expression of unique long (UL)-16 binding proteins-1 (ULBP-1), ULBP-2/5/6, ULBP-3, major histocompatibility complex (MHC) class 1-related (MIC)-A, MIC-B, CD48, CD80, CD86, CD112, CD155, Intercellular adhesion molecule (ICAM)-1, ICAM-2, HLA-E, HLA-F, HLA-A2, HLA-C, and the ligands to NKp30, NKp44, NKp46, and killer immunoglobulin-like receptor 3DS1 (KIR3DS1) by flow cytometry on CD4 T cells from 17 HIV-1 seronegative donors activated and infected with HIV. iCD4^+^ cells had higher expression of aNKR ligands than did unCD4. However, the expression of aNKR ligands on iCD4 where CD4 was downregulated (iCD4^−^) was similar to (ULBP-1, ULBP-2/5/6, ULBP-3, MIC-A, CD48, CD80, CD86 and CD155) or significantly lower than (MIC-B, CD112 and ICAM-2) what was observed on unCD4. Thus, HIV infection can be associated with increased expression of aNKR ligands or either baseline or lower than baseline levels of aNKR ligands, concomitantly with the HIV-mediated downregulation of cell surface CD4 on infected cells.

## 1. Introduction

Natural Killer (NK) cells are innate immune cells that mediate cytotoxic responses to stressed, virally-infected, and transformed cells [1]. NK cells can lyse target cells directly through the production of granzyme and perforin, or indirectly through antibody (Ab)-dependent mechanisms, through the production of cytokines and chemokines, and through activation of adaptive immune cells [2,3,4]. The induction and potency of these responses depend on the activation state of the NK cell, which is regulated by signals transmitted through both activating and inhibitory NK cell receptors (aNKR and iNKRs) [4,5]. Signals from iNKR often dominate by interrupting signals from aNKR, the consequences of which explain tolerance to self [6]. When signals from iNKR are interrupted, NK cell activation can occur if target cells express ligands to aNKR [1,4,5,7]. However, in vivo most cells co-express variable combinations of ligands to both NKR types and NK cell activation will depend on the integration of the number and strength of all activating and inhibitory signals transmitted.

NK cells express a variety of receptors. One of the major aNKRs is the C-type lectin natural-killer group 2, member D (NKG2D), which binds to a family of major histocompatibility complex class I (MHC-I)-like molecules that includes the human cytomegalovirus unique long (UL)-16 binding proteins (ULBP) and MHC-I related chain (MIC) proteins [8,9,10]. The expression of these ligands is induced by cellular stress and they are otherwise not found on the surface of healthy cells [11]. The other major family of aNKR comprises the natural cytotoxicity receptors (NCR) NKp30, NKp44, and NKp46 [8,12,13,14]. Although NCRs can bind membrane-associated heparan sulfate glycosaminoglycans and viral hemagglutinin, their cellular ligands remain incompletely identified [15,16,17,18,19].

Several additional receptors that contribute to NK cell activation by target cells have been identified. Two members of the CD2 subfamily of receptors—CD244/2B4 and the NK-T-B cell antigen (NTB-A)—can bind to CD48 and trigger NK cell cytotoxicity [20]. The leukocyte adhesion molecule DNAX accessory molecule-1 (DNAM-1) is widely expressed on NK cells and engages both CD112 (Nectin-2) and CD155 (poliovirus receptor) expressed on target cells [21,22,23]. DNAM-1’s ability to trigger NK cell responses requires co-expression of the lymphocyte function-associated antigen 1 (LFA-1), which interacts with the integrins intercellular adhesion molecules (ICAM)-1 and ICAM-2 on target cells to form the immunological synapse [24,25,26,27,28]. The T cell co-stimulatory B7 molecules CD80 and CD86 have also been shown to stimulate NK cell-mediated lysis through a mechanism that is currently undefined, but may depend on a CD28 variant expressed on NK cells [29,30,31,32].

While a subset of the classical MHC-I human leukocyte antigens (HLA)-A, -B, and -C are ligands to the inhibitory killer immunoglobulin-like receptor (KIR) family, the non-classical HLA-E and -F interact with aNKR [33]. HLA-E can signal through the inhibitory C-type lectin-like receptor NKG2A and the activating NKG2E and -C, all of which form heterodimers with CD94 [34,35,36]. HLA-F is a more recent addition to the family of ligands to aNKR and has been shown to stimulate degranulation and cytokine production through its binding to KIR3DS1 on NK cells [37,38].

It was previously shown that autologous HIV-infected CD4^+^ T cells are capable of activating NK cells ex vivo and that these activated NK cells will inhibit further viral replication [37,39,40]. The HIV accessory proteins negative regulatory factor (Nef) and viral protein U (Vpu) have well documented effects on expression of ligands to iNKR on the surface of infected cells. Specifically, Nef contributes to the internalization and downregulation of HLA-A and -B, abrogating inhibitory signaling through their cognate iNKR [41,42,43]. As NK cells are directed to kill cells with low expression of MHC-I antigens, the downregulation of HLA-A and -B by HIV should make infected cells more susceptible to NK cell lysis [44]. However, a third MHC-I antigen, HLA-C, and the non-classical HLA-E antigen remains expressed on the surface of infected cells, where they can engage inhibitory KIR2DL1, -2, and -3 and NKG2A/CD94, respectively, to inhibit NK cell activation [45]. However, more recent work suggests that Vpu-mediated downregulated of HLA-C can be observed with some primary clinical HIV isolates, although this is not observed with laboratory strains [46].

The influence of HIV on iNKR ligand expression and its impact on NK cell activation and subsequent antiviral responses has been well studied. The contribution of ligands to aNKR, however, is less well characterized. Here, we determined whether HIV-1 infection can modulate the expression profile of ligands to aNKRs on infected cells and identified aNKR ligands that are preferentially modulated by infection.

## 2. Materials and Methods

### 2.1. Ethics Statement

This study was approved by the Institutional Review Boards of the Comité d’Éthique de la Recheche du Centre Hospitalier de l’Université de Montréal and the Research Ethics Committee of the McGill University Health Centre-Montreal General Hospital. Informed consent was obtained from all study participants.

### 2.2. Study Population

This study included a total of 17 HIV seronegative donors. Eleven additional HIV seronegative donors were included in analyses of NK cell mediated inhibition of HIV replication in autologous infected CD4 T cells.

### 2.3. CD4^+^ T Cell Isolation

Peripheral blood mononuclear cells (PBMC) were isolated from blood by density gradient centrifugation (Ficoll-Paque; Pharmacia, Uppsala, Sweden) and cryopreserved in 10% dimethyl sulfoxide (Sigma-Aldrich, St. Louis, MO, USA) with 90% fetal bovine serum (FBS; Wisent, Inc., St. Bruno, QC, Canada). CD4^+^ T cells from 17 HIV seronegative individuals were positively selected from thawed PBMCs using a commercially available kit, as per manufacturer’s instructions (Stemcell Technologies, Inc., Vancouver, BC, Canada). The purity of the isolated cells was determined by flow cytometry and averaged 93.6 ± 3.5%.

### 2.4. HIV Infection

Following isolation, CD4^+^ T cells were incubated at 37 °C at a concentration of 2 × 10^6^ cells/mL in RPMI 1640 medium; 10% fetal bovine serum albumin (FBS); 2 mM L-glutamine; 500 IU/mL penicillin; 50 mg/mL streptomycin (R10; all from Wisent) supplemented with 100 IU/mL of recombinant human IL-2 (R10-IL-2; Chiron Corp., Emeryville, CA, USA) and activated with 1 μg/mL of phytohemagglutinin (PHA; MP Biomedicals, Santa Ana, CA, USA) for 24 hours. After activation, cells were washed and incubated at 37 °C in R10-IL2 for 72 hours. Following this incubation, at least 2 × 10^6^ cells were infected with the cysteine (C)-C chemokine receptor 5 (CCR5)-tropic HIV-1_JR-CSF_ (a kind gift from Dr. Galit Alter; Harvard University) at a multiplicity of infection of 0.01 and subsequently cultured at 37 °C at a concentration of 1 × 10^6^ cells/mL of R10-IL-2 for 7 days. Uninfected CD4^+^ T cells (unCD4) were cultured in parallel with their infected counterparts.

### 2.5. Antibody Staining and Acquisition

On day 7 post-infection, HIV-infected CD4^+^ T cells (iCD4) and unCD4 were stained with UV Live/Dead ^®^ Fixable Blue cell stain kit (Thermofisher, Waltham, MA, USA) and then with anti-CD3-BV785 (clone OKT3; BioLegend, San Diego, CA, USA) and anti-CD4-BUV737 (SK3; BD Bioscience, Missisauga, ON, Canada) in conjunction with monoclonal antibodies (mAbs) to the following cell surface markers, which were organized into 4 panels for the purpose of analysis: (Panel 1) ULBP-1-PerCP (170818), ULBP-2/5/6-APC (165903), ULBP-3-PE (166510), MIC-A-APC (159227), MIC-B-AlexaFluor 700 (236511; all from R&D Systems, Minneapolis, MN, USA), (Panel 2) CD48-PE (BJ40), CD80-BV421 (2D10), CD86-PE-Dazzle594 (IT2.2; all from BioLegend), CD112-AlexaFluor 700 (610603; R&D), CD155-PE-Cy7 (SKII.4; BioLegend), (Panel 3) ICAM-1-Pacific Blue (HCD54), and ICAM-2-PE (CBR-1C2/2; both from BioLegend). Cells were also stained with recombinant human IgG1 Fc chimeras NKp30-Fc, NKp44-Fc, and NKp46-Fc (all from R&D) as a part of Panel 3. A final HLA Panel included anti-HLA-E-PE-Cy7 (3D12; BioLegend) and 3D11, an unconjugated primary mAb against HLA-F (a kind gift from Dr. Daniel Geraghty, Fred Hutchison Research Institute, Seattle, WA, USA), HLA-A2-APC (BB7.2; eBioscience, San Diego, CA, USA), as well as DT9, an unconjugated anti-HLA-C mAb (EMD Millipore, Billerica, MA, USA). Briefly, cells were re-suspended in a 96-well V bottom plate (Sarstedt, Nümbrecht, Germany) at a concentration of 1 × 10^6^ cells per 100 μL of Dulbecco’s phosphate buffered saline (dPBS; Wisent) and stained with the Live/Dead reagent. TruStain FcX reagent (BioLegend) was used to minimize non-specific Fc receptor interactions and cells were stained separately with the Abs in Panel 1, 2, 3, and HLA for 30 min in the dark at room temperature (RT). Following staining, cells were washed, fixed, and permeabilized using the reagents in the Fix & Perm Cell fixation and permeabilization kit (Thermofisher), as per manufacturer’s instruction. Intracellular staining with anti-p24-FITC (KC57; Beckman-Coulter, Mississauga, ON, Canada) was used to determine the percentage of HIV-infected (iCD4), with unCD4 providing background values.

iCD4 and unCD4 stained with any of the chimeric human IgG1 Fc proteins, 3D11, or DT9 were included in the HLA panel and were prepared as above. They were incubated with the primary Ab on ice for 40 minutes. After washing, anti-HLA-C binding was detected with APC-conjugated polyclonal goat F(ab’)_2_ anti-mouse IgG secondary Ab (eBioscience) for 20 minutes on ice. All other primary Abs were detected using a polyclonal anti-human IgG (Fcγ-specific)-PE secondary Ab following the same protocol. Cells were then washed and stained with Abs to CD3, CD4, and intracellular p24 as previously described. After staining, all cells were washed and fixed with 2% paraformaldehyde (Santa Cruz, Dallas, TX, USA). Between 400,000 and 600,000 events were acquired on an LSRFortessa x20 (BD Biosciences, San Jose, CA, USA) flow cytometer within 24 h. Unstained, single stained control (CompBead; BD Biosciences, Mississauga, ON, Canada), fluorescence minus one, and secondary Ab alone controls were used for multi-colour compensation and gating purposes. Flow cytometric analysis was performed using FlowJo software version 10 (TreeStar, Inc., Ashland, OR, USA).

### 2.6. Inhibition of Viral Replication Assay

iCD4 cells were prepared as previously described and NK cells were isolated and stimulated with iCD4 according to a previously established protocol [40]. Briefly, iCD4s were plated at NK:iCD4 ratios of 10:1 or alone for 10 days in R10-IL-2. Cells were collected and stained with Aqua amine reactive fluorescent dye (Invitrogen, Burlington, ON, Canada) to determine viability and cell-surfaced stained with anti-CD3-APC-eFluor 780 (UCHT1; eBioscience) and anti-CD4-PE (RPA-T4; BD Biosciences, Mississauga, ON, Canada). p24 staining, acquisition, and analysis were performed as described above.

### 2.7. Statistical Analysis

Statistical analysis was performed using GraphPad Prism version 6 (GraphPad, San Diego, CA, USA). Wilcoxon tests or Friedman tests with Dunn’s post-test comparisons were used to compare the significance of differences in the frequency or mean fluorescence intensity (MFI) of a cell surface ligand between 2 or more than 2 matched groups, respectively. Spearman correlation tests were used to determine the significance of correlations between ligand expression and HIV infection. *p*-Values less than 0.05 were considered significant.

## 3. Results

### 3.1. CD4^+^ T Cell Infection Characteristics

**Figure 1:**
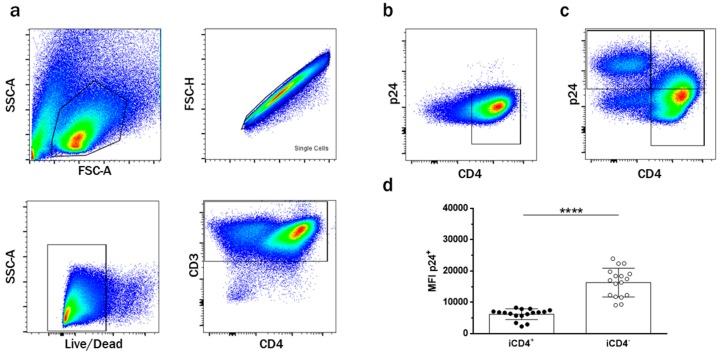
Gating strategy. (**a**) Gating strategy identifying live singlet CD3^+^ T lymphocytes. HIV-uninfected CD4^+^ T cells were identified from this gate (**b**); as were the different subsets of HIV-infected CD4^+^ T cells (**c**); (**d**) comparison of the mean fluorescence intensity (MFI) of p24 expression on HIV-infected p24^+^CD4^+^ (iCD4^+^) and HIV-infected p24^+^CD4^−^ (iCD4^−^) T cells (*n* = 17). A Wilcoxon test was used to determine significance of within subject differences for the indicated T cell subsets. Each data point represents a separate infection of CD4^+^ T cells isolated from one individual. Bar height and error bars represent the mean and standard deviation for the data set. Significant values are shown; **** *p* < 0.0001.

To investigate the expression of aNKR ligands on CD4^+^ T cells and the modulatory effects of HIV-1 infection on aNKR expression, we used 4 Ab panels specific for aNKR ligands. As the infection protocol requires culturing cells in IL-2-containing media, which can upregulate aNKR ligands, we cultured unCD4 T cells under the same conditions as an internal control to observe the effects of HIV infection on aNKR profiles irrespective of the effects of IL-2. The gating strategy in Figure 1a was used to identify T cells as single, live, CD3^+^ lymphocytes. As illustrated in Figure 1b,c, the expression of CD4 and intracellular p24—a measure of HIV-1 infection—was used to delineate 5 different T cell subsets. The first subset was HIV-uninfected p24^−^CD4^+^ T cells that had never been exposed to virus (unCD4s; Figure 1b). Similarly, from the population of cells that were cultured with HIV, we identified a subset of p24^−^CD4^+^ T cells that remained HIV-uninfected despite being in the same culture with infected cells, i.e., exposed uninfected (euCD4) (Figure 1c). Total infected p24^+^ T cells were defined as the iCD4^T^ subset. However, consistent with reports that the HIV-1 viral protein Nef can downregulate surface expression of CD4, two distinct subsets could be observed within iCD4^T^ [41,47]. We observed an HIV-infected p24^+^CD4^+^ T cell (iCD4^+^) subset and an HIV-infected p24^+^CD4^−^ T cell (iCD4^−^) subset in which cell-surface CD4 was downregulated (Figure 1b).

### 3.2. Comparison of the aNKR Ligand Expression Intensity on iCD4 and unCD4 T Cells

**Figure 2:**
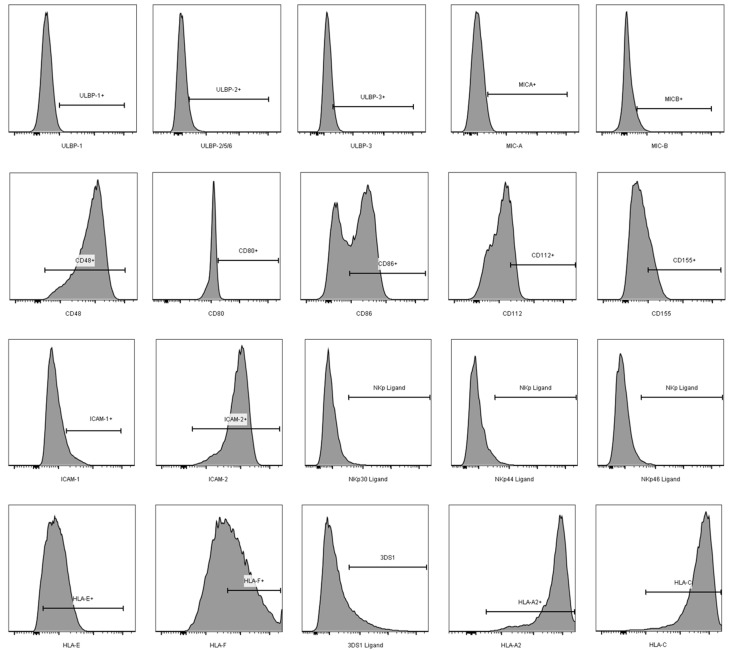
Histograms showing sample flow cytometry profiles for the ligands studied. Bars in each plot indicate the population expressing each aNKR, Gates were set using Unstained, single stained controls, fluorescence minus one, and secondary antibody alone controls.

**Figure 3:**
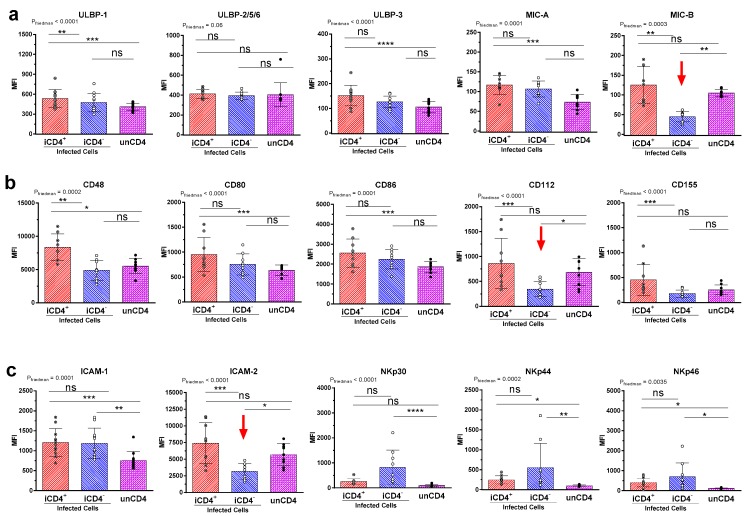
Mean fluorescence intensity (MFI) of aNKR ligand expressing T cell subsets. Comparison of the per-cell expression of Panel 1, 2, and 3 ligands to aNKR on HIV-infected p24^+^CD4^+^ T cells (iCD4^+^), p24^+^CD4^−^ T cells (iCD4^−^) and HIV-uninfected p24^−^CD4^+^ T cells (unCD4). Panel 1 (**a**) includes ULBP-1 (*n* = 11), ULBP-2/5/6 (*n* = 11), ULBP-3 (*n* = 11), MIC-A (*n* = 9), and MIC-B (*n* = 9); Panel 2 (**b**) includes CD48, CD80, CD86, CD112, and CD155 (*n* = 9, for all); Panel 3 (**c**) includes ICAM-1, ICAM-2, and the ligands to NKp30, NKp44, and NKp46 (*n* = 9, for all). The MFI of the expression of the indicated ligand is represented on the y-axis. Friedman (P_Friedman_) and Dunn’s post (*) tests were used to determine significance of within subject differences between data sets. Each data point represents T cells isolated from a separate individual. Bar height and error bars represent the mean and standard deviation for the data set. Significance values are shown over the bars linking 2 groups as * *p* < 0.05; ** *p* < 0.01; *** *p* < 0.001; **** *p* < 0.0001. Red arrows highlight ligands for aNKR expressed at a lower MFI on iCD4^−^ than unCD4 cells.

**Figure 4:**
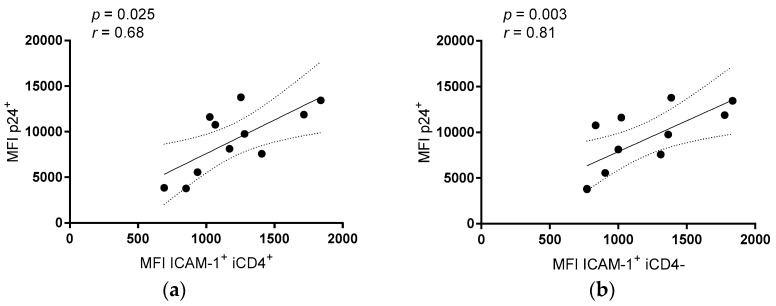
The per-cell expression of ICAM-1 is correlated with the MFI of p24 expression in HIV infected T cell subsets. Correlation of MFI of ICAM-1 expression with the MFI of p24 expression in (**a**) iCD4^+^, and (**b**) iCD4^−^ HIV infected T cell subsets. Each data point represents T cells isolated from a separate individual (*n* = 11). Dotted lines represent 95% confidence intervals. Significant values (*p*) and correlation coefficients (*r*) are shown.

**Figure 5:**
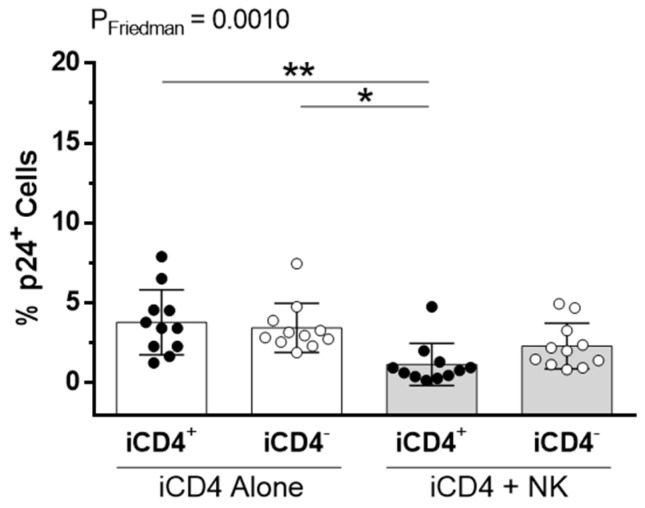
Natural killer (NK) cells preferentially target HIV-infected CD4 T cells that conserve cell-surface CD4 expression. The frequency of HIV-infected (p24^+^) cells within both iCD4^+^ and iCD4^−^ T cell subsets from iCD4 cultured alone (white bars) or co-cultured with NK cells (grey bars) for 10 days (*n* = 11). Friedman (P_Friedman_) and Dunn’s post (*) tests were used to determine significance of within subject differences between data sets. Each data point represents T cells isolated from a separate individual. Significant values are shown; * *p* < 0.05; ** *p* < 0.01.

It has been proposed that HIV iCD4^−^ cells are more productively infected than iCD4^+^ cells [47,48]. In line with this, we observed that iCD4^−^, compared to iCD4^+^ cells, have higher expression levels of p24 on a per cell basis (*p* ≤ 0.0001, paired *t*-test, Figure 1d). To characterize the extent of aNKR ligand modulation in HIV-1-infected CD4^+^ T cells, we assessed potential changes to the per-cell expression of the Panel 1, 2, and 3 aNKR ligands studied by measuring the mean fluorescence intensity (MFI) of aNKR ligand expression. Examples of histograms of the flow cytometry profiles of the aNKR ligands present on iCD4 that were investigated in this manuscript are shown in Figure 2. As seen in Figure 3, we observed that the MFI of ULBP-1, ULBP-3, MIC-A, CD48, CD80 and CD86 was significantly elevated on iCD4^+^, but not on iCD4^−^, compared to unCD4 (*p* ≤ 0.05, for all comparisons, Friedman with Dunn’s post-tests). We observed that the expression of CD86 on iCD4^+^ cells was significantly increased, relative to unCD4 (*p* = 0.002). In contrast, HIV infection was associated with a sustained increase in the MFI of ICAM-1 on iCD4^+^ and iCD4^−^ cells (*p* = 0.005 and 0.02, respectively). Furthermore, ICAM-1 was the only aNKR ligand studied whose intensity of expression positively correlated with the intensity of p24 staining in both iCD4 subsets (Figure 4). Interestingly, the three NCR ligands studied showed a unique pattern of expression whereby the MFI of NKp30, NKp44, and NKp46 ligands trended towards being higher on iCD4^−^ than on iCD4^+^ or unCD4 cells. In sum, we observed that HIV infection is associated with changes to the aNKR ligand expression profile that affects the MFI of ligand expression. Specifically, we found that compared to HIV-unCD4 the expression of ULBP-1, ULBP-3, MIC-A, CD48, CD80, CD86, ICAM-1 and the ligands for NKp44 and NKp46 was elevated on iCD4^+^, whereas expression of MIC-B, CD112, and ICAM-2 was reduced on iCD4^−^. We also observed that iCD4^+^ cells, rather than iCD4^−^ cells, were targeted by NK cells, following co-culture with iCD4 (Figure 5). It is plausible that the increased expression of ligands to aNKR that is observed on iCD4^+^ explains their significantly higher depletion levels (*p* = 0.0043, Friedman with Dunn’s post-test), as NK cells interacting with iCD4^+^ may be more highly activated.

### 3.3. Expression Profiles of HLA-E, HLA-F, HLA-A2, and HLA-C on iCD4 and unCD4

**Figure 6:**
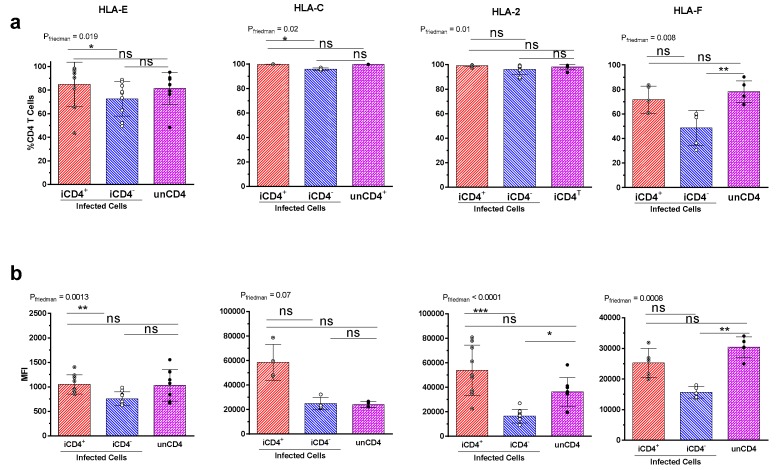
Expression profile of the human leukocyte antigen (HLA) molecules HLA-E, HLA-C, HLA-A2, and HLA-F on CD4^+^ T cell subsets. (**a**) The frequency of HIV-infected p24^+^CD4^+^ (iCD4^+^), p24^+^CD4^−^ (iCD4^−^) and uninfected (unCD4) T cells expressing HLA-E (*n* = 9), HLA-C (*n* = 4), HLA-A2 (*n* = 9), and HLA-F (*n* = 5); (**b**) the mean fluorescent intensity (MFI) of iCD4^+^, iCD4^−^ and unCD4 cells expressing the indicated ligands for the same study subjects as in (**a**). The percentage and MFI of cells expressing the indicated ligand is represented on the y-axis. Significant values are shown; * *p* < 0.05; ** *p* < 0.01; *** *p* < 0.001.

In addition to the aNKR ligands HLA-E and HLA-F—which are ligands for NKG2 receptors and KIR3DS1 respectively—we analysed the expression of two iNKR ligands, HLA-A2 and HLA-C (Figure 6). Expression of HLA-A2 was only assessed in iCD4 from persons typing positive for this HLA antigen. Although HIV Nef can downregulate the expression of HLA-A and B antigens on the surface of infected cells, cell-surface expression of HLA-E is maintained and the expression of HLA-C depends on the viral strain used [45,46]. While a lower frequency of iCD4^−^, compared to iCD4^+^, expressed HLA-E (*p* = 0.01, Dunn’s, Figure 6a) and did so with a lower intensity (*p =* 0.002, Dunn’s), the frequency and MFI of HLA-E expression on iCD4^+^ and iCD4^−^ cells did not differ significantly from that of unCD4, consistent with the observation that HLA-E is retained at the surface of bulk HIV-infected cells [45,49]. As HIV strains vary in their ability to down-modulate HLA-C we examined whether infection reduced HLA-C expression. We found that HIV infection did not significantly impact HLA-C expression on iCD4^+^ or iCD4^−^ cells compared to unCD4 (Figure 6a,b). While the frequency of HLA-A2^+^ iCD4^+^ and iCD4^−^ cells did not differ from that of unCD4 the MFI of HLA-A2 expression on iCD4^−^ was two-fold less than that of unCD4, though the difference did not quite achieve statistical significance (*p* = 0.0637) [50,51]. Overall, our findings support previous observations that the HIV viral protein Nef down-modulates expression of HLA-A, while leaving surface expression of HLA-E and HLA-C unmodulated. We also observed that the expression of HLA-F, the ligand for KIR3DS1, was downregulated on iCD4^−^, compared to unCD4 (*p* ≤ 0.004, for both comparisons). This is consistent with the recent observation that while HLA-F mRNA is increased overall in HIV-infected T cells, the expression of HLA-F is relatively decreased on iCD4^−^ compared to iCD4^+^ cells [37].

## 4. Discussion

Since the surface expression of HLA-A and -B antigens are downregulated over the course of HIV-infection, it is plausible that modulation of the expression of ligands to aNKRs on the surface of infected cells may have important regulatory effects on the NK cell responses they elicit. As such, elucidating HIV-induced regulation of the aNKR ligand expression profile on infected cells would help further characterize the NK cell stimulating potential of these cells. In this study, we report that HIV infection is associated with significant changes to the expression profiles of ligands to aNKRs. We observed two general patterns of HIV-induced ligand modulation. Specifically, we found that, compared to unCD4, the expression of ULBP-1, ULBP-3, MIC-A, CD48 CD80, CD86, ICAM-1 and the ligands for NKp44 and NKp46 was increased exclusively on iCD4^+^ cells, whereas iCD4^−^ cells had a reduced expression of MIC-B, CD112 and ICAM-2. Furthermore, ICAM-1 was found to be broadly upregulated on all HIV infected cell subsets examined.

The influence of HIV infection on the expression of some ligands to aNKR has been previously investigated in different infection models. While ULBP-1, ULBP-2/5/6, and ULBP-3 are rarely expressed on healthy primary CD4^+^ T cells, it is well established that infection with several laboratory strains of HIV can induce their surface expression [52,53,54,55]. In contrast, reports on HIV-mediated regulation of the expression of other NKG2D ligands, i.e., MIC-A and MIC-B, on CD4^+^ T cells are conflicting [52,54,55]. HIV infection induces the loss of surface expression of CD155 on the Jurkat T cell line, however, this could not be reproduced in bulk p24^+^ primary HIV_NL4-3_-infected T cells, which found CD155 to be modestly upregulated [56,57,58]. Infection of the human monocytic cell line U937 with primary isolates of an Indian HIV-1 subtype C induced the downregulation of CD80 and CD86 [59]. Finally, HIV infection has also been suggested to trigger the cell surface loss of CD48 on iCD4^+^ T cells [52]. In addition to these analyses of surface expression, an RNA microarray of primary human CD4^+^ T cells infected with the HIV-1-based reporter virus NL4-3 BAL-IRES-HSA found that ULBP-1, CD80, and ICAM-1 expression was upregulated, compared to mock-infected cells, consistent with our observations of increased surface expression of these ligands [60]. Moreover, the RNA expression profiles of these ligands did not differ between mock infected cells and uninfected bystander cells, which are equivalent to the euCD4 studied here [60].

The results presented here add to this body of work by including aNKR ligands not previously studied, including CD112 and ICAM-2. Additionally, in vitro HIV-infected CD4^+^ T cells can be easily divided into two distinct populations (iCD4^+^ and iCD4^−^), depending on their expression of CD4. In infected CD4 T cells, CD4 surface expression is reduced by internalization from the cell surface by Nef, which targets the receptor to lysosomes for degradation [41,42,43,47]. HIV Env can interfere with the transport of CD4 to the cell surface and Vpu targets CD4 for degradation in proteasomes [61,62,63]. Regardless, most of the existing literature assessed the aNKR phenotype of bulk p24^+^ infected cells, regardless of CD4 expression. It has, however, been postulated that iCD4^+^ and iCD4^−^ differ in the burden and timing of HIV infection and we also observed that the intensity of staining for the viral capsid protein p24, which serves as a marker of infection, differed significantly between these two subsets. HIV-infected CD4^+^ T cells have been shown in vitro to activate NK cells, which in turn can block viral replication [39,40]. The ligands studied here and their engagement of aNKR likely contribute to anti-HIV NK cell responses and differences in the expression of these ligands on both subsets of infected cells may alter their ability to activate NK cells. To address this knowledge gap, we devised a gating strategy that allowed us to examine the expression profiles of aNKR ligands on both iCD4^+^ and iCD4^−^ cell subsets and compare them to those of euCD4 and unCD4. As hypothesized, aNKR ligand expression did differ on iCD4^+^ and iCD4^−^, with iCD4^+^ expressing higher levels of 9 out of the 15 aNKR ligands studied. We also observed that iCD4^+^ cells were preferentially targeted by NK cells, leaving iCD4^−^ virtually untouched, compared to the co-culture of NK cells with unCD4. Together, these findings suggest that HIV-infected T cells expressing CD4 likely constitute better stimuli for NK cell activation, as they express higher levels of the aNKR ligands studied here. Their preferential depletion suggests that they are also targeted more effectively by NK cell than are iCD4^−^ cells.

Additionally, we observed that these two infected T cell subsets differed phenotypically from unCD4, further emphasizing the ability of HIV infection to modulate aNKR ligand expression. We found that HIV-induced modulation was ligand specific. The expression of the stress ligands ULBP-1, ULBP-3, and MIC-A, CD48, CD80 and CD86 was increased on iCD4^+^, but their expression on iCD4^−^ cells remained at levels comparable to those observed on unCD4. On the other hand, HIV-infection did not upregulate the expression of MIC-B, CD112 and ICAM-2 on iCD4^+^, compared to unCD4, but reduced expression of these ligands on iCD4^−^ to levels below those observed on unCD4. Whether this is a Nef-dependent phenomenon is at present unknown. Nef is one of the HIV gene products responsible for the downregulation of CD4 on these iCD4 and has previously been demonstrated to influence certain aNKR ligands on a variety of HIV-infected cell types [41,43,47]. It is well supported that Nef is required for the selective downregulation of the MHC-I molecules HLA-A and HLA-B and can also block the trafficking of newly synthesized MHC-I proteins to the cell surface [45,50,64,65,66]. Nef is also instrumental in inducing the loss of cell surface CD155 in T cell lines and CD80 and CD86 in the human monocytic cell line U937 [56,59]. While HIV infection has been reported by others to be associated with increased expression of ULBP-1, ULBP-2, and ULBP-3, Nef expression is associated with the downregulation of NKG2D ligands and expression of ULBP-1, ULBP-2 and MIC-A increases more dramatically in cells infected with Nef-deficient virus [52,55]. These findings are consistent with our observation that the increased expression of ULBP-1, ULBP-3, and MIC-A in iCD4^+^ is lost in parallel with CD4 downregulation. Considering that down-modulation of aNKR ligands may thus protect HIV-infected cells from NK cell targeting, our observation that expression of MIC-B, CD112 and ICAM-2 is reduced on iCD4^−^ to levels below those observed on unCD4 is of particular interest. It is therefore plausible that the downregulation of these ligands to aNKR on the surface of iCD4^−^ may confer a survival advantage to these cells by dampening their ability to activate NK-cell mediated anti-HIV responses. Nevertheless, the regulation of these ligands by viral proteins is a complex process and, at this time, the specific contribution of these aNKR ligands to NK activation and antiviral NK cell responses and the ways in which infection might influence them remains unclear.

Notably, we found that ICAM-1 expression was consistently increased across all infected cell subsets, regardless of CD4 expression. ICAM-1 and ICAM-2 are adhesion molecules that both contribute to the formation of the immunological synapse between an NK and target cell, which facilitates NK cell activation [26,27,28]. However, expression of ICAM-1—but not ICAM-2—was also shown to be required for efficient dendritic cell-mediated HIV transmission and increased infectivity by almost 10-fold [67,68]. It is possible that the consistent upregulation of ICAM-1 on all infected cell subsets contributes to improved viral transmission and may therefore be favorable to viral propagation, despite its effects on NK cell activation.

These findings and our observations that HIV induces significantly different aNKR ligand expression phenotypes on iCD4^+^ and iCD4^−^ cells, suggest that the activity of the viral proteins such as Nef, Vpu and possibly others may underlie the regulation of aNKR ligand expression in HIV-infected CD4^+^ T cells. It is also possible that iCD4^+^ and iCD4^−^ may be at different stages of infection, as the time from infection with respect to the presence or production of Nef and Vpu varies in iCD4 T cells [69]. However, the exact relationship between the timing of viral protein production and CD4 downregulation remains incompletely understood and additional work characterizing the activity of viral proteins in both iCD4^+^ and iCD4^−^ and its association with aNKR ligand expression will be required further exploration.

Although our study focuses on these ligands in the context of NK cell activation, it is important to consider the contribution of several of the ligands studied here to inhibitory signaling. For example, HLA-E, the expression of which was not significantly modulated by HIV infection, can signal through both inhibitory (NKG2A) and activating (NKG2E and -C) members of the NKG2x/CD94 family of NK cell receptors [34]. HLA-C, which we and others have observed remains expressed on CD4^+^ T cells infected with lab-adapted virus, engages the inhibitory killer immunoglobulin receptors KIR2DL1, -2, and -3, limiting NK cell activation by infected cells [45].

Activation of NK cells both in vitro and in vivo is a complex process that depends on the balance and potency of the different activating and inhibitory signals transmitted upon interaction with a potential target cell. While our findings provide the foundation for an improved understanding of the way HIV infection can impact NK cell activation through the modulation of the expression of aNKR ligands, the complete picture of how these changes contribute to activating signaling and how this might integrate with potential inhibitory signaling requires further investigation.

## 5. Conclusions

There is a lack of consensus on how to analyze HIV-infected CD4 T cells (iCD4) by flow cytometry and iCD4 are largely treated as one homogenous population. In this manuscript, we directly compare the expression of aNKR ligands on both subsets of infected T cells and demonstrate that iCD4 comprise two distinct subsets that can be differentiated by their expression or not of cell-surface CD4. We show that these two subsets differ phenotypically. Our observations also offer insights into the ligand receptor interactions that may be important in governing individual NK cell responses and activation. We believe that this research will assist future studies of NK cell activation and will contribute to harnessing the therapeutic potential of NK cells in the context of HIV infection and beyond.
